# Magnetically Controlled On‐Demand Switching of Batteries

**DOI:** 10.1002/advs.202000184

**Published:** 2020-02-28

**Authors:** Jiaqian Zhang, Xiaohui Zhu, Mengqi Zeng, Lei Fu

**Affiliations:** ^1^ College of Chemistry and Molecular Sciences Wuhan University Wuhan 430072 China; ^2^ The Institute for Advanced Studies (IAS) Wuhan University Wuhan 430072 China

**Keywords:** batteries, electrical transition, liquid metal, magnetic actuation, on‐demand switching

## Abstract

The integration of stimuli‐responsiveness into energy storage devices has become an attractive way to manage the operation of devices. Current stimuli approaches (light, chemical, and temperature) require transparent windows and specific systems, or are subject to the tolerant temperature of batteries, hampering their widespread applications. Herein, a fast and reversible on‐demand switching of batteries, which is realized by incorporating a magnetic control component, is reported. The component is capable of undergoing a reversible transition between electrical conduction and insulation over 500 cycles, showing superior cycling stability. Batteries with this component internally incorporated can retain excellent electrochemical performance in a wide potential window at normal conditions. More importantly, this approach can manage the operation of batteries in light of human requirements. The battery can shut down within 0.11 s of applying a magnetic field and rapidly resume a normal battery function under the magnetic field, showing an excellent response speed. Notably, this on‐demand switching behavior in batteries can be repeated over 25 times, excelling most reported switching batteries. The design combines fast and repeatable characteristics without sacrificing electrochemical performance, providing possibilities in advancing the development of smart electronics.

## Graphical abstract

On‐demand switching of batteries is realized by means of actuating magnetic liquid metal marble under the magnetic field. Such on‐demand switching can control the operating state of devices in the light of human requirements. Notably, this on‐demand switching behavior demonstrates the capacity of repeatability and fast response time, providing new insights and further applications for devices.

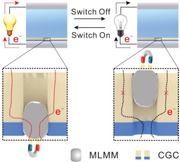

With the ever‐merging development on smart electronics, managing the operation of devices by conventional approaches can no longer cater to human requirements.^1^, ^2^ Exploring more intelligent methods is currently of great urgency. Recently, the integration of stimuli‐responsiveness into energy storage devices is a fascinating way with the prospect of switching devices by external physical or chemical stimuli.^3^ Typical thermal‐responsive materials, including thermal expansion polymer,^4^ sol‐gel transiting materials,^5^ and positive temperature coefficient materials^6^, ^7^ have been demonstrated to shut down batteries with the increasing temperature. However, the transition temperature of thermal‐responsive materials is difficult to tune, meanwhile the development rate of new thermal‐responsive materials cannot keep up with the demands for practical applications. For redox‐responsive materials, redox‐switchable nanovalve is capable of realizing the power switch in a concentration cell.^8^ Nevertheless, the chemical‐based approaches require a specific system, which greatly limits their widespread applications. More importantly, all the above attempts fail to establish a dynamic user–device interaction to meet versatile demands. Namely, users cannot discretionarily control the operating state of devices at any time. On this account, a unique design that can control the operating state of devices in the light of human requirements and simultaneously has no performance compromise represents an on‐demand switching concept, which is urgent to be integrated into energy storage devices.

Exploring suitable materials and developing a highly integrated design are desirable to realize an on‐demand switching. Recently, liquid metals (LMs) are an emerging class of materials possessing an extraordinary series of properties such as high conductivity, deformability, fluidity, and reconfiguration capability.^9^, ^10^, ^11^, ^12^, ^13^, ^14^ Owing to the polarizable surface and weak liquid–liquid attraction, LMs can easily combine with ferromagnetic materials.^15^ The magnetic LMs afford concomitantly the remarkable properties of ferromagnetic materials and LMs, which can integrate a series of emerging functionalities, especially magnetic actuation and rheology capacities. Owing to the contactless manipulation, strong penetrability, and fast response of magnetic field, magnetic LMs provide an available strategy to realize the on‐demand switching.^16^, ^17^, ^18^ As is reported, when confined to a narrow channel, magnetic LM can easily pass through it depending on its deformability.^19^ In addition, researchers have confirmed that LM marble can be actuated on various substrates by means of coating the LM with hydrophobic particles, showing the remarkable properties including low friction, nonstick property, and anti‐impact capacity.^20^ Thus, the magnetic‐responsiveness of LM marble is expected to realize on‐demand switching of batteries.

Herein, we present a magnetic control component to realize the on‐demand switching of batteries. The component is capable of exhibiting reversible electrical transitions between electrical conduction and insulation under the applied magnetic field. After experiencing the electrical transition over 500 cycles, no obvious damage in this component is observed. Batteries internally incorporated component remain excellent electrochemical performance in a wide potential window at normal conditions, and it can promptly shut down as on‐demand under the applied magnetic field. It can also resume the normal function immediately in the light of human requirements. The on‐demand behavior can be switched 25 times without the sacrificial electrochemical performance, exhibiting a more superior cyclicity than previously reported switching devices. Beyond that, such on‐demand switching behavior is highly sensitive to the magnetic field. The fast response time is 0.11 s, showing an excellent response speed. This is the first time that a fast and reversible on‐demand switching of batteries without sacrificing the battery performance is realized under the magnetic field, delivering new insights into smart electronics and promoting further applications.

It is well‐known that electrochemical reactions in batteries involve both ionic and electronic transportations.^7^ In this regard, the batteries can instantly shut down upon the impeded transportation of ions or electrons. To implement the concept of on‐demand switching of batteries, a magnetic control component is designed to control the electronic transport. The component is placed between the electrode and cell shell, where the cathode is taken as an example (**Figure** **1**a). The schematic illustration of the mechanism in on‐demand switching of batteries is proposed in Figure 1b. The component constructs from conductive gradient channel (CGC) and magnetic liquid metal marble (MLMM). Generally, MLMM is located at the bottom of CGC, connecting the upper conductive layer with a cell shell. The highly conductive MLMM provides pathways for electronic transport, ensuring the normal operation of batteries. After applying a suitable magnetic field to the anode side, the MLMM moves away from the cell shell, meanwhile, the component becomes electrically insulating. The pathways of electronic transport are cut off, which results in the shutdown of the battery. Conversely, when the magnetic field applied to the cathode side, the MLMM moves back to the original position and the component becomes electrically conductive. Thus, the battery resumes cycling on account of the unimpeded electronic transport. Such an electrical transition arising from a magnetically actuating process is highly reversible, endowing the on‐demand switching of batteries with repeatable character.

**Figure 1:**
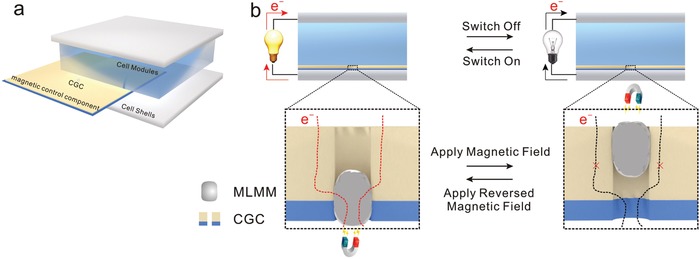
a) Schematic illustration of a battery with a magnetic control component. The yellow module represents Cu foam. The blue module represents the PDMS film. b) Schematic illustration of on‐demand switching of batteries under a magnetic field. The modified battery operates normally when MLMM is placed at the bottom of CGC. Applying a magnetic field to the Cu foam side, the MLMM is driven, and the component becomes insulating and shuts down the battery. Applying a reversed magnetic field, the MLMM is moved back to the original position and resumes the normal cycling.

Scanning electron microscopy (SEM) image of CGC displays that a cylindrical channel with a diameter of 900 µm is located in a copper/polydimethylsiloxane (Cu/PDMS) conductive gradient layer (**Figure** **2**a). The graphite coating layer is covered in the insides walls of CGC, providing a smooth channel for the magnetic actuation of MLMM. The cross‐sectional image exhibits that the PDMS film and Cu foam adhere tightly, where the total thickness of them is ≈1.2 mm (Figure 2b). As shown in Figure 2c, the as‐prepared MLMMs possess a uniform size with a diameter of 1000 µm (the MLMM was prepared by dispersing iron (Fe, 32.09 ± 2.97 µm, Figures S1 and S2, Supporting Information) particles in eutectic alloys of gallium and indium (EGaIn), and then the magnetic LM‐based droplets are rolled around in a graphite powder bed for 5 s). SEM was conducted to further reveal the microscopic morphology of MLMM. As displayed in Figure 2d, the surface of magnetic LM‐based droplet is coated uniformly with graphite. This result is attributed to the strong adhesion between the oxidized layer of LM and graphite, which ensures that graphite adheres tightly on the surface of LM.^21^ It is worth noting that LM/Fe still maintains a smooth and liquid surface, confirming that the introduction of Fe has no compromise on the liquid property of LM (Figure S3, Supporting Information). Further, energy‐dispersive X‐ray spectroscopy (EDX) mapping reveals the even distributions of Ga, In, Fe, and carbon, implying that there is no aggregation of Fe particles in the LM matrix due to the weak liquid–liquid attraction of LM (Figure 2e). This observation indicates that Fe particles can be turned to well‐aligned chains, offering a uniformly magnetic force distribution in the direction of the magnetic field during the actuation process. To further reveal the structure of LM/Fe, X‐ray diffraction (XRD) analysis was conducted on LM/Fe, pure LM, and pure Fe. A broad characteristic peak of 35° is observed in pure LM, corresponding to amorphous EGaIn (Figure 2f). Meanwhile, the characteristic peak is also detected in LM/Fe, proving the liquid characteristic of LM/Fe. This result is consistent with the SEM analysis. Besides that, no alloy peak containing Fe is detected in LM/Fe except the peak of pure Fe (45°), which indicates that Fe particles dispersing into LM are elemental rather than alloy state.

**Figure 2:**
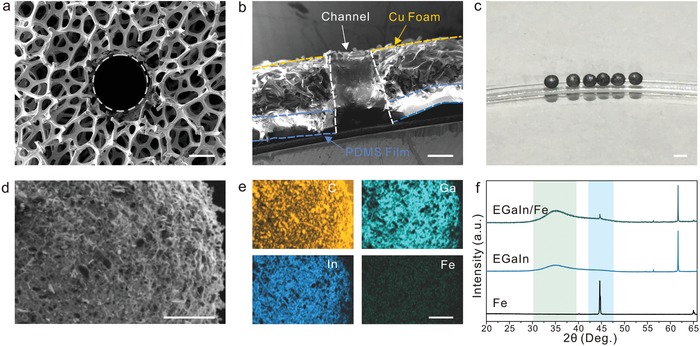
a) Top view and b) cross‐sectional SEM images of the as‐prepared CGC. Scale bar, 400 µm. c) Photograph of the as‐prepared MLMM using the PDMS template method. Scale bar, 1 mm. d,e) SEM image of the MLMM with Fe content of 20 wt%, and related EDX elemental mapping of the same location. Scale bar, 100 µm. f) XRD patterns of LM/Fe with Fe content of 20 wt%, and the patterns of pure GaIn and pure Fe for reference.

To investigate the actuating behavior of MLMM, in situ optical microscopy (OM) tests were performed (**Figure** **3**a–e; Movie S1, Supporting Information). The diameter of the designed CGC (900 µm) is smaller than MLMM (1000 µm). Hence, owing to the deformability of MLMM, the shape of the MLMM located in the bottom of CGC is closed to ellipse initially, suggesting a good electrical contact between the CGC and cell shell (Figure 3a). By applying a magnetic field of 500 gauss (Gs) to the Cu foam side, the MLMM as a whole moves along CGC (Figure 3b,c), revealing that the actuating behavior is a nondestructive process. The oxide layer provides a mechanical stability, stabilizing the LM marble to retain morphologies and preventing the encapsulated Fe particles from escaping.^22^ And the graphite coating layer further endows MLMM with nonwettable characteristic.^23^ Afterward, we apply a magnetic field of 500 Gs to the side of the PDMS film, the MLMM can immediately return to the PDMS film side along the initial path (Figure 3d,e), confirming that the actuating behavior is reversible. Notably, based on liquid properties, the size of MLMM can be distributed in an adjustable range to realize on‐demand switching behavior, where the size of MLMM ranges from 900 to 1200 µm (Figure S4 and Table S1, Supporting Information). Furthermore, the actuating performance can also be tuned by varying Fe contents and magnetic fields. It is found that LM/Fe begins to lose liquid state when Fe content is in excess of 20 wt%, which gives a clear indication of rigidity increase as Fe particles are packed more and more heavily (Figure S5, Supporting Information). Hence, directing with Fe contents below 20 wt%, detailed analyses on the actuation of MLMM under the various Fe contents and magnetic field are performed in Figure S6 (Supporting Information). The actuating distance increases when the MLMM subjected to a higher magnetic field, but the increasing trend of the higher Fe content is inclined to slow down gradually. The decrease in trend is ascribed to the fact that the MLMM with a higher Fe content has reached to the Cu foam side quickly. Therefore, the Fe content of 20 wt% is selected to guarantee a superior electrical transition. In addition, under a constant magnetic field of 2000 Gs, the velocity of actuation can be improved with the increase of Fe content. As Fe content of 20 wt%, the maximally actuating velocity can reach to 9.6 mm s^−1^, providing a fast response velocity for on‐demand switching of batteries.

**Figure 3:**
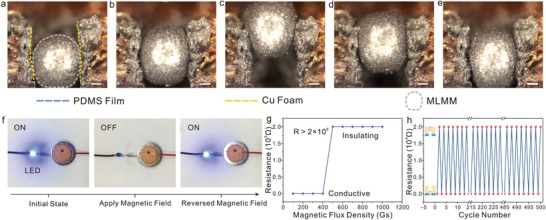
In situ OM visualization and conductivity variation of the actuating process. Taking the MLMM with Fe content of 20 wt% as an example. a) Optical image of MLMM located at the bottom of the PDMS film, corresponding to the initial state of the magnetic control component. b,c) The MLMM moves along CGC by applying a magnetic field of 500 Gs to the Cu foam side. d,e) The MLMM moves back to the bottom of the magnetic control component along the initial path at applying a magnetic field of 500 Gs to the PDMS film side. Scale bar, 200 µm. f) Demonstration of the electrical transition behavior of the component. g) The resistance of component as a function of the magnetic field. h) The electrical transition between electrical conduction and insulation for 500 cycles upon changing the magnetic field.

Based on the above actuating behavior, Figure 3f reveals the electrical transition of the magnetic control component under the actuating process. A light‐emitting diode (LED) is connected to a component in the circuit and lights up at initial. The LED shuts down instantly after applying a magnetic field of 500 Gs to the Cu foam side, indicating that the component becomes electrically insulating. When applied to a magnetic field of 500 Gs to the PDMS film side, the LED lights up again, revealing the component returns to electrically conductive. These results exhibit that the actuating process of MLMM can induce the electrical transition of the component. To further explore it, the resistance dependence on the magnetic field was measured. The resistance value is negligible at initial and then the resistance sharply increases by ≈8 orders after applying a magnetic field of 500 Gs to the Cu foam side (Figure 3g). By measuring the actuating distance under the key magnetic flux densities (200, 400, 500, 700, and 1000 Gs), the electrical transition process in resistance can be completely reflected in virtue of the position of the MLMM (Figure S7, Supporting Information). Moreover, the electrical transition of component is not only merely reversible, but also repeatable. Figure 3h exhibits the 500 cycles electrical transition of the component under the magnetic field, indicating that the magnetic actuation of MLMM has long‐term stability. Surprisingly, the MLMM after 500 cycles remains integrated, showing no noticeable ruptures (Figure S8, Supporting Information). Meanwhile, the cycled MLMM has a similar morphology to that of the pristine MLMM, verifying the integrity of the MLMM after long‐term cycling. Such a stable, reversible, and repeatable actuation process is especially important for on‐demand switching of batteries.

To verify the feasibility of on‐demand switching in batteries, we assembled lithium||LiFePO_4_ (Li||LFP) batteries modified by the magnetic control component for comparison. In view of the strong penetrability of the magnetic field, the component placed between the cathode and cell shell can also be controlled (**Figure** **4**a). Then MLMM with Fe content of 20 wt% was selected to obtain a fast response velocity of switching. Cyclic voltammetry (CV) curve of the modified battery exhibits perfectly overlap with normal battery, indicating that the component possesses high compatibility with conventional batteries (Figure S9, Supporting Information). The capacity retention of the modified battery is also consistent with the normal battery after 150 cycles, further confirming the addition of component is no sacrifice of the electrochemical performance of the battery. Based on it, the on‐demand switching behavior is explored under the magnetic field. As shown in Figure 4b, the modified battery displays a well‐defined CV curve with redox peaks at 3.8/3.1 V initially. Due to a slightly impeded effect in cell modules, a stronger magnetic field is required to on‐demand switch batteries. Hence, a magnetic field of 1700 Gs is applied to the Li electrode side. The battery shows a straight CV profile without redox peaks, in which there is a shutdown behavior occurred (Movie S2, Supporting Information).^6^ This phenomenon results from the impeditive electronic transport between the LFP electrode and cell shell. Subsequently, the redox currents increase and the CV curve overlaps with the initial state once applied a magnetic field of 700 Gs to the LFP electrode side. The battery resumes normal cycling under a weaker magnetic field because the actuation of MLMM is mainly affected by the different thickness of cell modules. What's more, such on‐demand switching behavior can also be verified in electrochemical impedance spectroscopy (EIS) tests. As shown in Figure 4c, the EIS spectrum of the modified battery consists of a semicircle (high frequency) and a sloping line (low frequency) (*R* = 27 Ω) under the normal conditions. After applying a magnetic field to the Li electrode side, the EIS spectrum reveals a nearly straight line. The modified battery is similar to a pure resistor, confirming that the battery undergoes a shutdown behavior. In virtue of a reversed magnetic field of 700 Gs, no obvious difference is observed between the initial and resumed impedance. These results demonstrate that the magnetically on‐demand switching of batteries is a highly reversible process.

**Figure 4:**
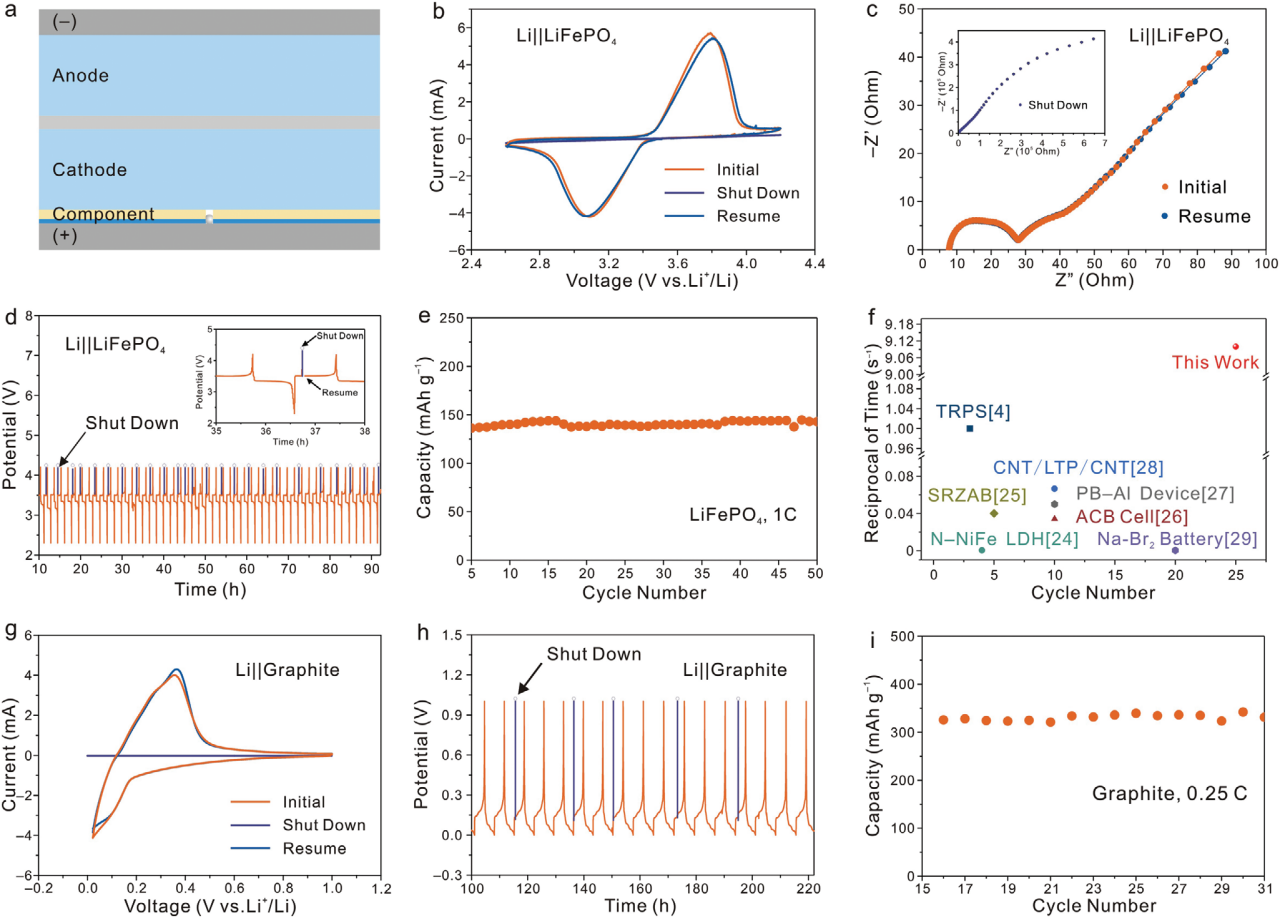
Electrochemical performance and on‐demand switching behavior of batteries. a) Schematic of battery modified by a magnetic control component. b,c) CV curves and EIS spectra of the modified Li||LFP battery during the switching process. d) Cyclic tests on on‐demand switching behavior. The inset displays the enlarged voltage profile of switching behavior. e) The capacity retention of the modified Li||LFP battery after 25 switching times. f) Comparison of response time between the published literatures and our work. g) CV curves of Li||graphite battery during the switching process. h) The charge/discharge voltage profile during the on‐demand switching process. i) The capacity retention of Li||graphite battery after five switching times.

As expected, the on‐demand switching can be repeated many times. As displayed in Figure 4d, once a magnetic field of 1700 Gs is applied to the Li electrode side, the modified battery shuts down instantly owing to the sharply increasing voltage. On the contrary, it can resume and keep stable cycling after providing a magnetic field of 700 Gs to the LFP electrode side. The inset in Figure 4d shows the modified battery recovers at a pristine open potential, which suggests almost no leakage current during the switching process.^6^ Surprisingly, the on‐demand switching behavior of the modified battery can be reproduced over 25 times and the battery still possesses 95% capacity retention (Figure 4e). Subsequently, the battery was dissembled for observing the morphology of MLMM after cycling. The MLMM still keeps integrated without any breakage and can be reversibly actuated under the magnetic field (Figures S10 and S11, Supporting Information). This demonstrates that the on‐demand switching is highly repeatable and stable. Furthermore, we measured the response time with respect to the magnetic field with different magnetic flux densities (Figure S12, Supporting Information). The minimum magnetic flux density required to switch battery is 1700 Gs. And we can clearly see that the switching time decreases with the increase of the magnetic flux density to 1900 Gs. With further increasing magnetic flux density, the switching time sharply decreases and reaches a saturation point at 2300 Gs, after which we do not observe a significant decrease in the switching time. Thereinto, the shortest switching time is ≈0.11 s. By comparing with the reported literatures (Figure 4f),^4^, ^24^, ^25^, ^26^, ^27^, ^28^, ^29^ the magnetically on‐demand switching process owns significant advantages over the switching rate in the reported switching devices.

To further evaluate the on‐demand switching behavior of batteries at a low potential window, we chose graphite‐based batteries to perform cycling performance tests of on‐demand switching under the magnetic field. Similarly, CV curves and cycling performance of the modified Li||graphite battery are consistent with the normal battery, which suggests that the component can keep greatly compatible even at low potential (Figure S13, Supporting Information). The similarly on‐demand switching is also observed in the modified Li||graphite battery. As shown in Figure 4g, the large redox currents of the modified Li||graphite battery decrease to zero after applying a magnetic field of 1700 Gs to the Li electrode side, whereas the redox currents resume back to the initial state after applying a magnetic field of 700 Gs to the graphite electrode side. This on‐demand switching process can be repeated over the changing magnetic field. The voltage profiles demonstrate that the modified Li||graphite battery switching for five times still remains high stable, where no clear leakage current is observed (Figure 4h). Moreover, the modified Li||graphite battery delivers a capacity of 328 mAh g^−1^ and has no any degradation during the switching process (Figure 4i). Above all, the on‐demand switching behavior of batteries is highly compatible, fast, and controllable with no sacrificing electrochemical performance.

In summary, we have presented a fast, reversible, and repeatable on‐demand switching of batteries, which can manage the operation of batteries as on‐demand. This is realized by internally incorporating a magnetic control component into batteries. The magnetism‐based approach combines contactless manipulation with strong penetrability, breaking the intrinsic restrictions of batteries with regard to the other stimuli and offering a fast response time for on‐demand switching behavior. Batteries modified by this component can remain excellent electrochemical performance in a wide potential window. In the light of human requirements, it can shut down optionally and resume over 25 times, showing a superior cyclicity. Notably, the lowest response time of on‐demand switching is 0.11 s, exhibiting a higher responsive performance than previously reported switching batteries. Our design provides a combination of fast response, high repeatability, and excellent electrochemical performance, ensuring to manage the operation of batteries on demand. Besides, further development of the on‐demand switching in electrostatic discharge protection will offer plenty of opportunities for more practical applications in smart electronics. Hence, this novel design opens a new pathway into the functional batteries field.

## Conflict of Interest

The authors declare no conflict of interest.

## Supporting information

Supporting Information

Supplemental Video 1

Supplemental Video 2
