# Supplementary material for: Magnetically Controlled On‐Demand Switching of Batteries
Source: Adv Sci (Weinh). 2020 Feb 28;7(8):2000184. doi: 10.1002/advs.202000184 (PMC7175272; doi:10.1002/advs.202000184)
Supplement: Supplementary file 1 — Supporting Information [file ADVS-7-2000184-s001.pdf]

Copyright WILEY-VCH Verlag GmbH & Co. KGaA, 69469 Weinheim, Germany, 2020.

ADVANCED SCIENCE
Open Access

Supporting Information

for *Adv. Sci.*, DOI: 10.1002/advs.202000184

Magnetically Controlled On-Demand Switching of Batteries

*Jiaqian Zhang, Xiaohui Zhu, Mengqi Zeng, and Lei Fu**

Supporting Information

Magnetically Controlled On-Demand Switching of Battery

By *Jiaqian Zhang#, Xiaohui Zhu#, Mengqi Zeng, and Lei Fu**

#These authors contributed equally to this work.

Experimental Section

Fabrication of magnetic liquid metal marbles: EGaIn composed of 78.4 wt.% Ga (99.999%, Shanghai Minor Metals Co., Ltd) and 21.6 wt.% In (99.999%, Shanghai Minor Metals Co., Ltd), was obtained by mixing and heating in a vacuum drying oven at 200 ºC for 4 h. Firstly, the as-prepared EGaIn was added into 1M hydrochloric acid ethanol solution to remove the oxide layer. Subsequently, Fe particles (400 mesh, 98%, Aladdin Co., Ltd) with the certain mass ratios were taken out of the glove box to suspend in 100% ethanol. Then the Fe particles solution was added into 1M hydrochloric acid ethanol solution cotaining the EGaIn, followed by manually stirring with the speed of 2 r/s for 20 min. There have some points should be noticed. Fe particles shoule be stored in the glove box to restrain oxidation and the addtion of Fe particles should be divide into several times to prevent the excessive reaction between the Fe particles and hydrochloric acid. Later, the as-prepared LM/Fe was washed with ethanol several times and dried at room temperature for 12 h.

Fabrication of magnetic liquid metal marbles: The MLMM was prepared by a poly(dimethylsiloxane) (PDMS) template method. The PDMS template owns holes with fixed size, which was purchased from Suzhou Nabang Photoelectric Technology Co., Ltd. Firstly, the PDMS template was coated with graphite powder (purchased from Acros Organics) to prevent the adhesion between the EGaIn/Fe and PDMS. And then the excess graphite powder should be removed from the PDMS template. Secondly, a glass slide was used to force the EGaIn/Fe to fill into the holes. And the excess EGaIn/Fe must be wiped out to ensure the

uniform size of MLMM. Finally, the above EGaIn/Fe droplets were rolled around in a graphite powder bed for 5 s and the MLMM could be obtained. In the virtue of the PDMS template with different holes, the size of MLMM can be distributed in an adjustable range.

Fabrication of magnetic control component: PDMS elastomer was obtained by mixing with prepolymer and crosslinker (10/1, v/v). Then, the PDMS was blade-coated on the Cu foil substrate and solidified in a drying oven at 80 ºC for 6 h. After stripping from the Cu foil, a free-standing PDMS film can be obtained. A conductive gradient layer consisted of the PDMS film and Cu foam, both of which were glued together by the uncured PDMS elastomer. Subsequently, CGC can be obtained through a mechanical drilling. The inside wall of the CGC was coated with graphite slurry (prepared by mixing the graphite powder and 4% polyvinylidene fluoride (PVDF), mass ratio of 6:1) and then heated in a vacuum drying oven at 80 ºC for 12 h. Finally, the as-prepared MLMM was placed directionally in the bottom of CGC by the magnet.

In-situ OM measurements: The actuating process of MLMM was observed by an optical microscope (Keynece, VH-Z100R). The component and magnet ($Nd_2Fe_{14}B$, Shenzhen Xinhongchang Magnetic Materials Co., Ltd, N35 grade) were adhered to the ends of the propeller, respectively. After positioning the magnet into an appropriate location, the magnet and component were separated by a magnetism shielding device (zr-2000, thickness: 2.5 mm, purchased from Zhaorong soft magnetic materials Co., Ltd. of Shenzhen City). The magnetic shielding material can avoid the effects of $Nd_2Fe_{14}B$ magnet. The actuating process can be captured by OM in real time once pull away the magnetic shielding material. Similarly, based on the in-situ OM video of the actuation process, the response time and actuating distance can be recorded to obtain the response velocity.

Material characterizations: SEM studies were characterized by ZEISS Merlin Compact SEM. The structure of LM/Fe was investigated by XRD (SmartLab 9kW) using the Cu Kα radiation, where the scanning scope ranged from 20º to 80º. The actuating process of MLMM

was observed by an optical microscope. DLS measurement was performed on Malvern Panalytical Co. Ltd (MS3000).

Battery fabrication and testing: All the electrochemical tests were conducted by the half-cells (standard CR2032 cell). 1 M $LiPF_6$ was dissolved in ethylene carbonate/diethylcarbonate (1/1, v/v) as electrolyte. Cathode materials were prepared by LFP, carbon black and PVDF (mass ratio of 8:1:1). The magnetic control component was incorporated into the battery between the LFP electrode and the cathode shell. CV and EIS measurements were tested on an electrochemical workstation (CHId604E, Chenhua, Shanghai). Galvanostatic charge/discharge cycling was conducted on a multichannel battery tester (LAND CT 2001A, Wuhan LAND Electronics Co., Ltd.). The switching behaviors were measured by a home-made propeller. The magnet was fixed on one end of the propeller and the battery was fixed on another end of the propeller. By measuring the magnetic field intensity as a function of the distance between battery and magnet, precise control of magnetic field intensity can be achieved. Hence, a stated distance between magnet and battery was carried out to measure the response of the battery to the magnetic field.

Figure S1. The size distribution of Fe particles measured by DLS. The mean and standard deviation of the Fe particle size distribution are calculated using log-normal fits on the histogram plot. The mean value of the Fe nanoparticle size is 32.09 μm with the standard deviation value of 2.97 μm.

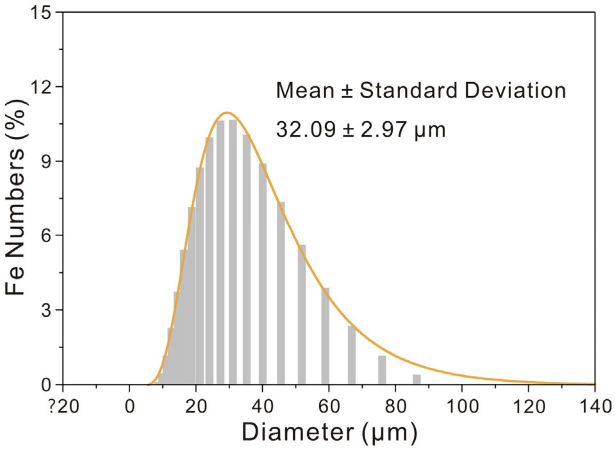

Figure S2. SEM image of Fe particles. The Fe particles are irregular shapes but the size distributions are relatively small. The small size distribution is coincident with the result of DLS. Scale bar, 100 μm.

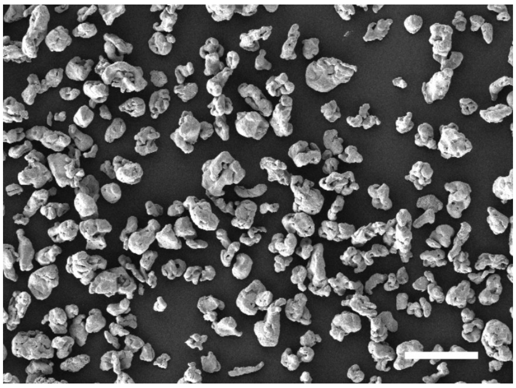

Figure S3. SEM image of LM/Fe with Fe content of 20 wt.%. Scale bar, 10 μm.

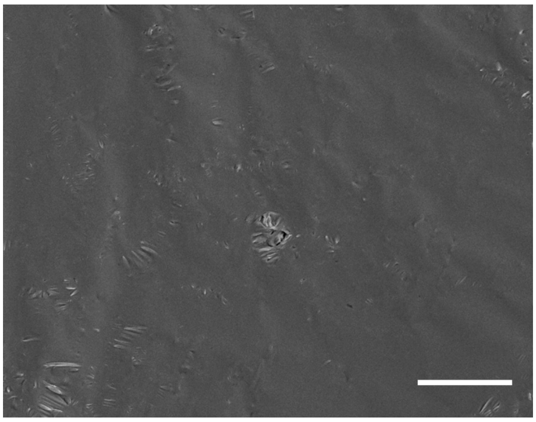

Figure S4. In-situ OM visualization of the actuating process via MLMMs with different sizes under the applied magnetic fields. a,c,e,g,i) Optical images of MLMMs with different sizes (including 800, 900, 1000, 1100, 1200 μm) locating at the bottom of the PDMS film, corresponding to the initial state of magnetic control component. b,d,f,h,j) Optical images of MLMMs with different sizes moving along CGC by applying a magnetic field to the Cu foam side. Scale bar, 500 μm.

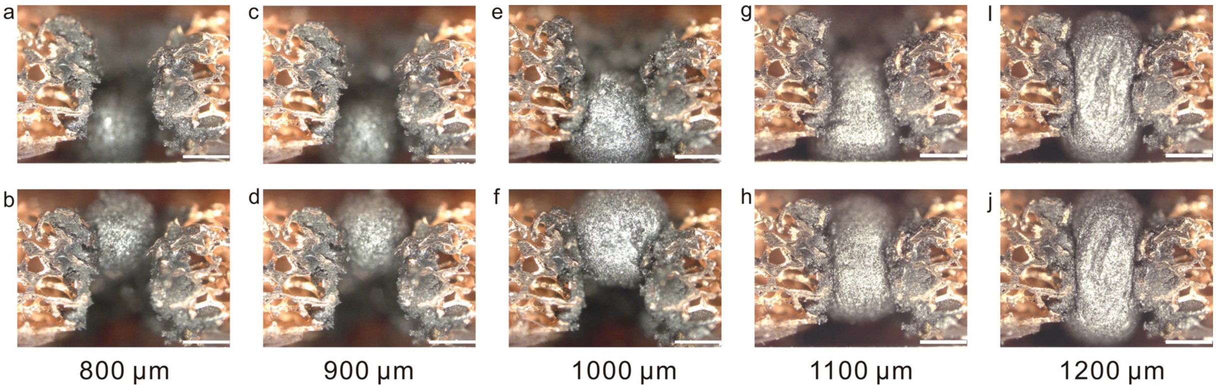

Figure S5. Digital photographs of LM droplets with various Fe contents. The results indicate that LM could maintain liquid properties with Fe (400 mesh) content below 20 wt.%.

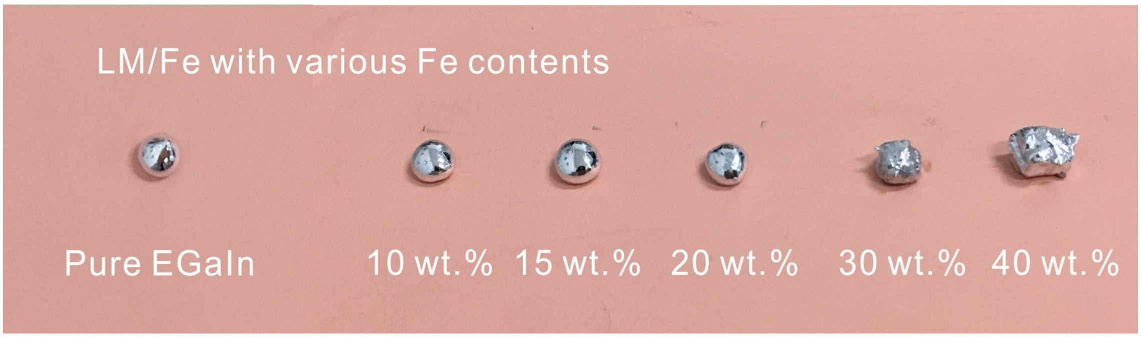

Figure S6. a) The actuating distance dependence of magnetic flux density with varying Fe contents. b) Average actuating velocity as a function of Fe contents.

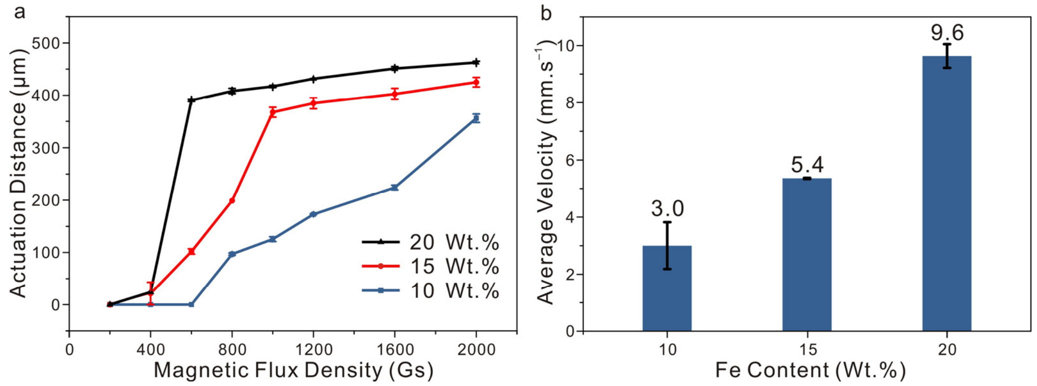

Figure S7. In-situ OM visualization under the magnetic field with the different magnetic flux density including 0 Gs, 200 Gs, 400 Gs, 500 Gs, 700 Gs, 1000 Gs. Scale bar 200 µm.

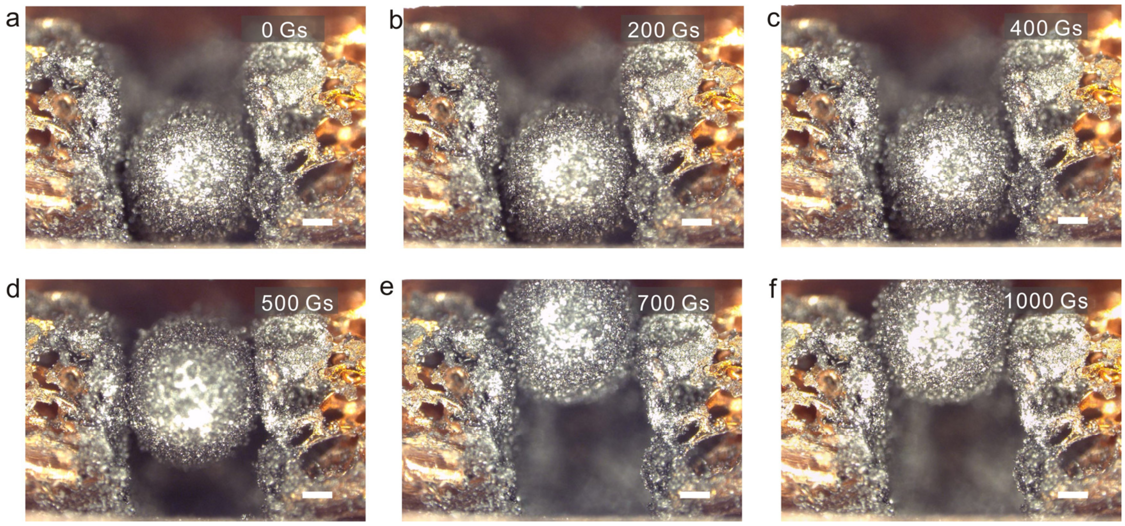

Figure S8. Optical images of the MLMM a) before cycling and b) after electrical transitions over 500 times. Scale bar, 500 μm.

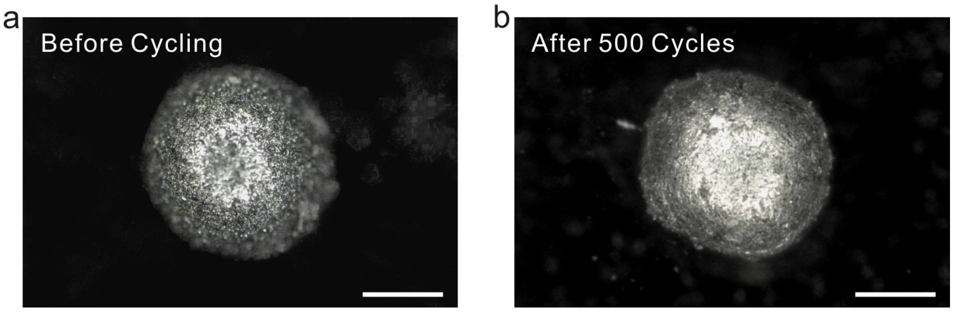

Figure S9. a) CV tests of the normal battery (Li||LFP) and the modified battery at a rate of 0.5 mV s^{-1}. b) Cycling performance of normal battery and the modified battery in the potential window of 2.3 – 4.2 V. They show similar specific capacity (~ 150 mA h g^{-1}).

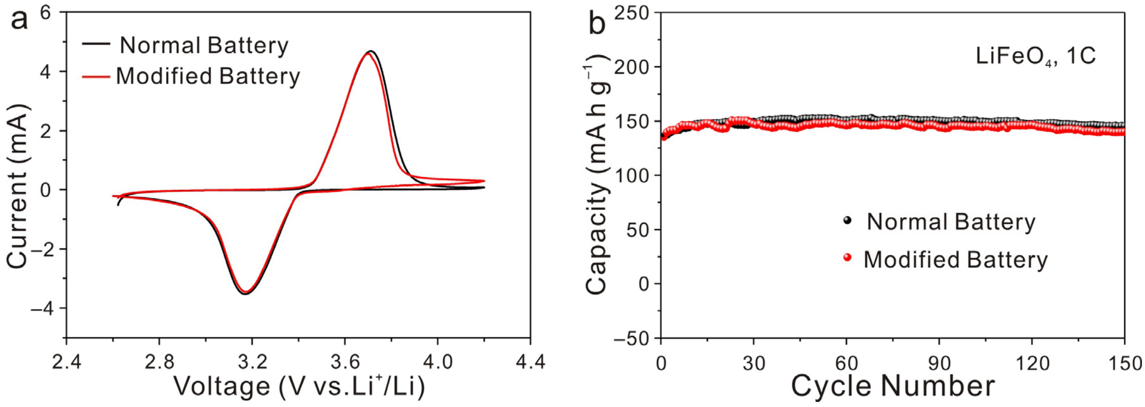

Figure S10. Digital image of MLMM after 25 cycles of switching in the battery in Li||LFP battery. Scale bar, 500 μm.

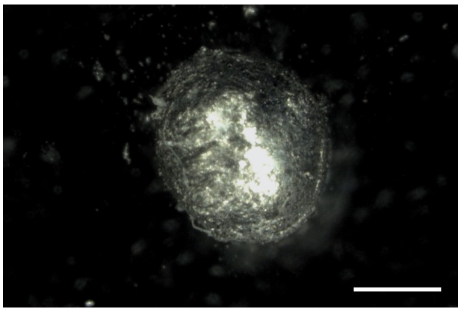

Figure S11. Magnetic actuation process of the MLMM after 25 cycles in the battery. a) OM image of the MLMM in the CGC, corresponding to the switching state. b) OM image of the MLMM in the CGC, corresponding to the shutdown state. c) OM image of the MLMM in the CGC, corresponding to the resuming state. Scale bar, 500 μm.

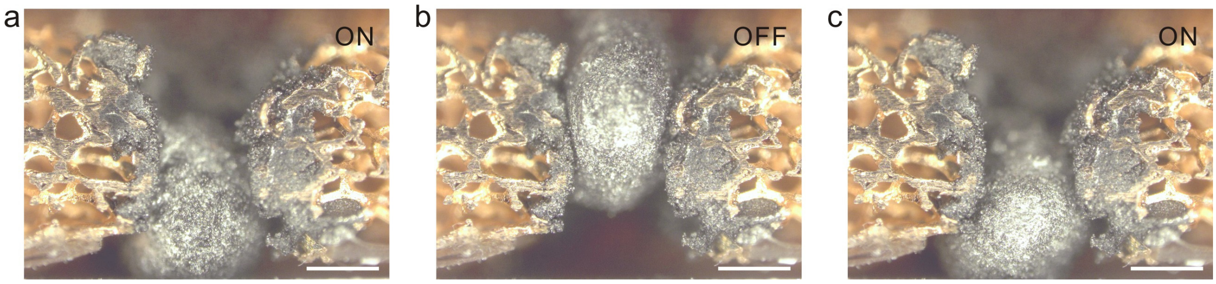

Figure S12. Plot of response time versus applied magnetic flux density for the battery modified by a magnetic control component.

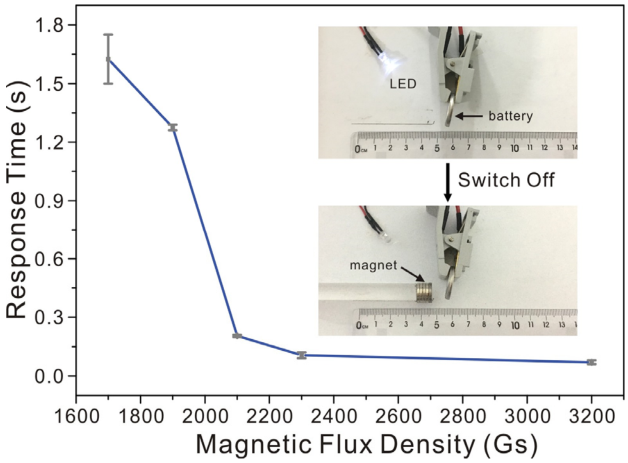

Figure S13. a) CV tests of the normal battery (Li||graphite) and the modified battery at a rate of 0.5 mV s^{-1}. b) Cycling performance of normal battery and the modified battery in the potential window of 0 – 1 V. They showed similar specific capacity (~ 328 mA h g^{-1}).

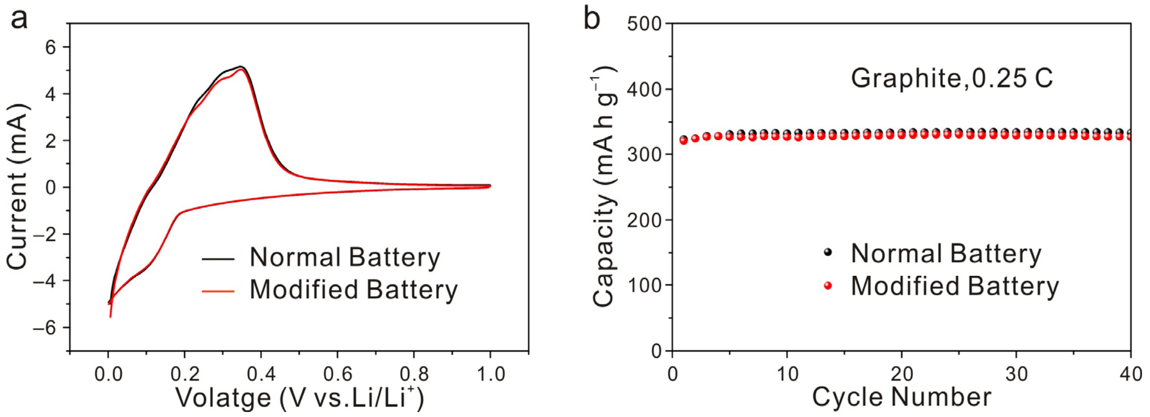

Table S1. The electrical transition behavior of the magnetic control component of MLMM with different sizes under the normal and shutdown conditions.

MLMM Diameter (μm)	Normal Resistance (Ω)	Shutdown Resistance (Ω)
800	1.2×10^6	—
900	48.1	352
1000	3.2	$> 2 \times 10^8$
1100	1.7	$> 2 \times 10^8$
1200	1.4	3.0

Supporting Movies and Legends:

Movie S1: In-situ OM visualization of MLMM under the actuating process.

Movie S2: Demonstration of the on-demand switching of batteries.
